# A Chondroblastic Osteosarcoma of the Patella: A Case Report

**DOI:** 10.7759/cureus.40777

**Published:** 2023-06-22

**Authors:** Mehak Kuchhal, Yashwant Lamture

**Affiliations:** 1 Surgery, Jawaharlal Nehru Medical College, Datta Meghe Institute of Higher Education and Research, Wardha, IND

**Keywords:** mri, knee joint, swelling, patella, chondroblastic osteosarcoma

## Abstract

Osteosarcoma is a form of bone cancer that can originate in any bone throughout the body, although it is most commonly detected in the long bones of the arms and legs. Chondroblastic osteosarcoma, a rare variation of osteosarcoma, is associated with a greater likelihood of recurrence and metastasis. The term "chondroblastic" indicates that certain portions of the tumor have undergone changes, resembling a connective tissue called cartilage. In this report, we present an exceptional case of osteosarcoma affecting the patella. A 54-year-old patient sought consultation at our outpatient clinic, reporting a 10-year history of swelling in the left leg. Magnetic resonance imaging revealed the presence of a large, diverse mass lesion located near the proximal region of the left leg. A Tru-cut biopsy confirmed the presence of chondroblastic osteosarcoma.

## Introduction

Osteosarcoma is a prevalent form of bone cancer commonly found in adolescents. This type of cancer originates from bone-forming cells and leads to the production of abnormal bone tissue. Treatment typically involves surgical removal of the tumor, while limb-saving procedures are often employed by doctors to maintain functionality. Additionally, chemotherapy is necessary to eradicate any hidden cancer cells that may have spread during the initial diagnosis. Osteosarcoma is the most common solid bone cancer, affecting approximately 2-3 individuals per 106,000 [1,2].

Limb-saving surgeries have proven highly effective in treating most cases of bone cancer without necessitating amputation. This achievement is largely attributed to improved chemotherapy administered before and after the surgical procedure. Consequently, more individuals diagnosed with osteosarcoma have a higher chance of longer survival. By combining imaging techniques, chemotherapy, and surgery, doctors can save the limb in approximately 70-85% of bone cancer cases. As a result, patients can expect to achieve long-term survival rates of approximately 60% [3,4].

Limb-sparing surgery demonstrates positive outcomes in cancer treatment, offers functional benefits, and contributes to psychological well-being. Nevertheless, patients undergoing prosthetic implantation following surgery may not experience significant physical improvement due to poor femur lesions, which can result in disease recurrence and decreased overall survival rates. Osteosarcoma frequently affects the proximal tibia, with roughly 75% of cases occurring near the knee. Removing osteosarcoma in this region using the broad incision concept often leads to bone and patellar tendon loss. Additionally, the growth plate in the tibia does not return to its normal state after inserting a distal femoral prosthesis [5]. These issues contribute to a higher risk of relapse, and complications related to prosthetic implantation following limb-sparing surgery are quite common. These complications include infections, joint component wear, dislocations, prosthesis breakage, fatigue, aseptic loosening, and long-term fractures.

## Case presentation

**Figure 1:**
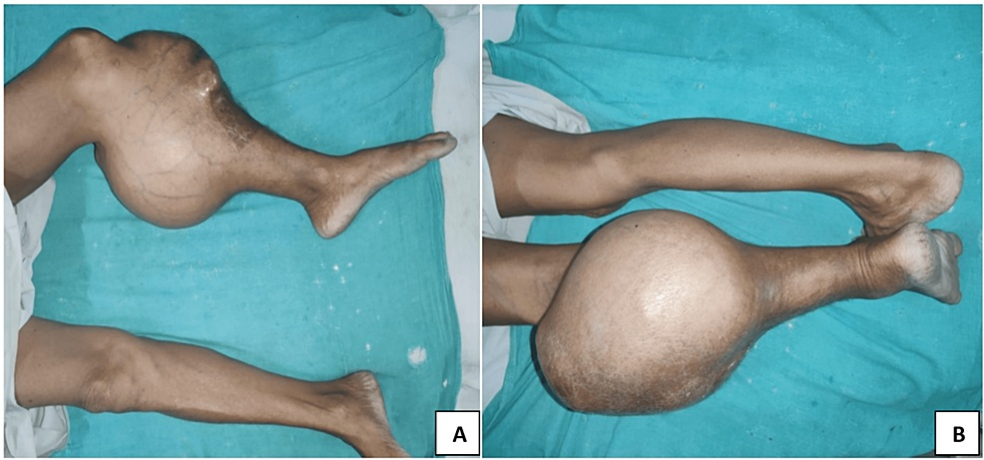
Clinical presentation of left leg chondroblastic osteosarcoma of the patella

A 54-year-old female patient presented at our outpatient clinic with a history of swelling in her left leg for the past 10 years. The patient reported that about a decade ago, she first noticed a swelling measuring approximately 3 by 2 centimeters below her knee on the left leg. Over time, the swelling has gradually progressed and now measures a massive 40 by 30 centimeters. The onset of the swelling was slow and gradual, with progressive nature. Upon physical examination, a swelling of 40 by 30 centimeters was observed on the patient's left leg, located just below the popliteal fossa (Figure 1).

**Figure 2:**
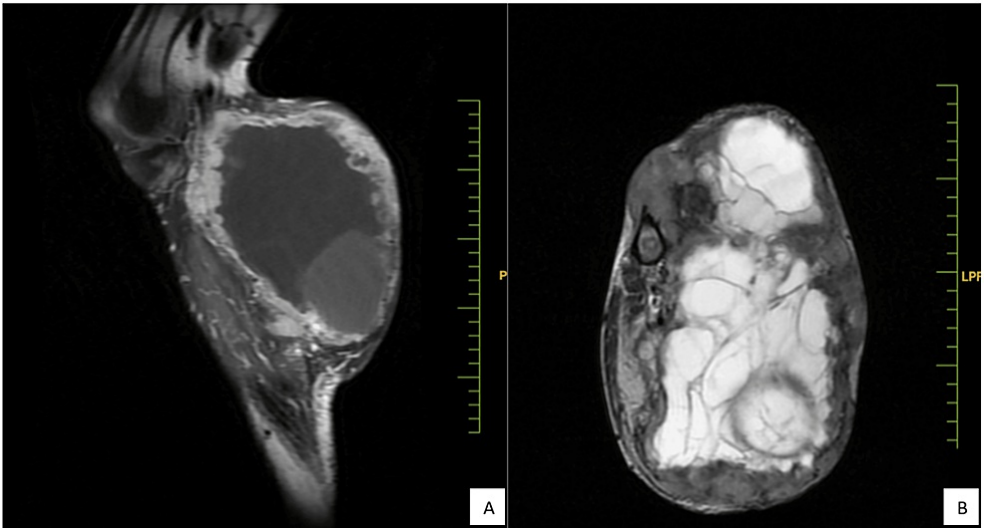
MRI left leg (A) sagittal view and (B) axial view MRI: magnetic resonance imaging

The swelling is not tender and has a firm consistency. There is a local increase in temperature, and the swelling does not exhibit fluctuation. Dilated veins are present over the swollen area, but the slip sign is negative. Magnetic resonance imaging (MRI) revealed the presence of a large and diverse mass lesion in the upper left leg (Figure 2). The mass lesion, which originates from its patella, has destroyed the fibula. The tibia is also affected by the mass. The extent of tibial involvement measures approximately 178 mm. The mass itself has dimensions of approximately 215 by 124 mm. On T1-weighted imaging, the mass appears as an area of low signal intensity, while on T2-weighted imaging, it shows high signal intensity within the tibia. The remaining portion of the mass exhibits heterogeneous signal intensity, characterized by large cystic components and internal septations. A section taken from the biopsy of the soft tissue tumor on the left leg confirmed the presence of osteogenic sarcoma (chondroblastic type).

## Discussion

Osteosarcoma is a form of bone cancer characterized by the development of osteoid or bone produced by cancer cells. It typically affects the ends of long bones near the knee, with the distal femur and proximal tibia being common sites. However, the patella (kneecap) is rarely affected, as indicated by a study conducted at the Mayo Clinic, which found only one patellar osteosarcoma out of 1649 osteosarcoma cases [2]. In the patella, benign tumors are more prevalent than malignant tumors, with a ratio of 73% to 27% [1]. The patella and the epiphysis of long bones share a similar ossification pattern. Giant cell tumors and chondroblastomas, considered epiphyseal tumors, are most frequently observed in the patella [1,3-6]. Malignant neoplasms of the patella include hemangioendothelioma, lymphoma, metastasis, and osteosarcoma [2].

Individuals with osteosarcoma typically experience pain, particularly during movement. They may initially mistake it for sprains, arthritis, or growing pains. Occasionally, a previous injury may be present. Although rare, osteosarcoma can lead to bone fractures, with telangiectatic osteosarcoma being more prone to this occurrence [7-9]. Discomfort in the lower extremity can result in a limp.

The presence of edema may vary depending on the size and location of the lesion. Systemic symptoms such as fever and nocturnal sweats are not common. Respiratory symptoms caused by lung metastases are uncommon and usually indicate significant lung involvement. Other symptoms are infrequent, as metastases to other locations are rare. Arthrodesis may be required for treatment. Metastases occur in only 15-20% of patients, predominantly affecting the lungs, although other bones can also be involved. The presence of osteosarcoma in multiple bone sites at the time of diagnosis may suggest the presence of multifocal sclerosing osteosarcoma [10]. Symptoms in individuals with osteosarcoma are typically limited to where the cancer originated, manifesting as a lump, pain, stiffness, swollen lymph nodes, or difficulty breathing. The exact cause of osteosarcoma is unknown, but certain factors can increase the risk of developing it [11].

Osteosarcoma often develops during periods of rapid bone growth, such as puberty. This could explain its higher incidence in certain larger animals, including dogs, and its tendency to occur near the growth plate of long bones. Certain genetic conditions can also elevate the risk of osteosarcoma, including those affecting the eyes (retinoblastoma), bones (Paget's disease, fibrous dysplasia, enchondromatosis, hereditary multiple exostoses), or the entire body (Li-Fraumeni syndrome with a TP53 mutation, Rothmund-Thomson syndrome) [8,10].

Various other bone conditions can resemble osteosarcoma. Some cancers include Ewing's sarcoma, neuroectodermal primitive tumors, rhabdomyosarcoma, fibrosarcoma, and chondrosarcoma. Others are non-cancerous conditions, such as histiocytosis, osteomyelitis, stress fractures, hematoma, chondroblastoma, chondromyxoid fibroma, osteochondroma, osteoblastoma, bone cysts, and giant cell tumors. Laboratory tests play a crucial role in assessing the impact of chemotherapy on the body by measuring organ function before, during, and after treatment. X-rays alone cannot definitively determine whether a bone lesion is osteosarcoma. Osteosarcoma bone lesions can be solely osteolytic (about 30% of cases), osteoblastic (about 45% of cases), or a combination of both. Occasionally, the periosteum elevates and forms a Codman triangle, which can indicate osteosarcoma [7,8]. A "sunburst" appearance may result from tumor extension through the periosteum.

## Conclusions

Chondroblastic osteosarcoma of the patella is an uncommon and highly aggressive tumor that necessitates prompt identification and treatment. Fine-needle aspiration (FNA) cytology can serve as a valuable tool for preoperative diagnosis, particularly when combined with clinical and radiological findings. The optimal treatment approach for this tumor remains a subject of debate, but limb-sparing surgery involving wide resection and reconstruction may offer superior functional outcomes compared to patellectomy. Additionally, chemotherapy using the MAP regimen may contribute to improved survival rates for patients with this tumor. Further research is necessary to assess the long-term prognosis and recurrence rates associated with chondroblastic osteosarcoma of the patella.

## References

[1] Mercuri M, Casadei R (2001). Patellar tumors. Clin Orthop Relat Res.

[2] Devaney K (1996). Dahlin's bone tumors: general aspects and data on 11,087 cases. Am J Surg Pathol.

[3] Kransdorf MJ, Moser RP Jr, Vinh TN, Aoki J, Callaghan JJ (1989). Primary tumors of the patella. A review of 42 cases. Skeletal Radiol.

[4] O'Mara JW Jr, Keeling J, Montgomery EA, Aaron AD (2000). Primary lesions of the patella. Orthopedics.

[5] Bhagat S, Sharma H, Bansal M, Reid R (2008). Presentation and outcome of primary tumors of the patella. J Knee Surg.

[6] Okada K, Sato K, Abe E, Kataoka Y, Miyakoshi N, Ishikawa N, Sageshima M (1994). Case report 858: postradiation osteosarcoma of the patella. Skeletal Radiol.

[7] Hudson M, Jaffe MR, Jaffe N (1990). Pediatric osteosarcoma: therapeutic strategies, results, and prognostic factors derived from a 10-year experience. J Clin Oncol.

[8] Mialou V, Philip T, Kalifa C (2005). Metastatic osteosarcoma at diagnosis: prognostic factors and long-term outcome - the French pediatric experience. Cancer.

[9] Nagarajan R, Clohisy D, Weigel B (2005). New paradigms for therapy for osteosarcoma. Curr Oncol Rep.

[10] Bacci G, Ferrari S, Bertoni F (2000). Long-term outcome for patients with nonmetastatic osteosarcoma of the extremity treated at the istituto ortopedico rizzoli according to the istituto ortopedico rizzoli/osteosarcoma-2 protocol: an updated report. J Clin Oncol.

[11] Bacci G, Briccoli A, Longhi A (2005). Treatment and outcome of recurrent osteosarcoma: experience at Rizzoli in 235 patients initially treated with neoadjuvant chemotherapy. Acta Oncol.

